# Distinct pattern of cerebral blood flow alterations specific to schizophrenics experiencing auditory verbal hallucinations with and without insight: a pilot study

**DOI:** 10.18632/oncotarget.23631

**Published:** 2017-12-23

**Authors:** Rixing Jing, Jiangjie Huang, Deguo Jiang, Xiaodong Lin, Xiaolei Ma, Hongjun Tian, Jie Li, Chuanjun Zhuo

**Affiliations:** ^1^ National Laboratory of Pattern Recognition, Institute of Automation, Chinese Academy of Sciences, Beijing, China; ^2^ University of Chinese Academy of Sciences, Beijing, China; ^3^ Department of Psychological Medicine, Wenzhou Seventh People's Hospital, Wenzhou, Zhejiang Province, China; ^4^ Department of Psychological Medicine, Tianjin Anning Hospital, Tianjin, China; ^5^ Department of Psychiatric Neuroimaging Laboratory, Tianjin Anding Hospital, Tianjin Mental Health Center, Teaching Hospital of Tianjin Medical University, Tianjin, China

**Keywords:** schizophrenia, auditory verbal hallucination, insight, cerebral blood flow

## Abstract

Schizophrenia is associated with widespread and complex cerebral blood flow (CBF) disturbance. Auditory verbal hallucinations (AVH) and insight are the core symptoms of schizophrenia. However, to the best of our knowledge, very few studies have assessed the CBF characteristics of the AVH suffered by schizophrenic patients with and without insight. Based on our previous findings, Using a 3D pseudo-continuous ASL (pcASL) technique, we investigated the differences in AVH-related CBF alterations in schizophrenia patients with and without insight. We used statistical parametric mapping (SPM8) and statistical non-parametric mapping (SnPM13) to perform the fMRI analysis. We found that AVH-schizophrenia patients without insight showed an increased CBF in the left temporal pole and a decreased CBF in the right middle frontal gyrus when compared to AVH-schizophrenia patients with insight. Our novel findings suggest that AVH-schizophrenia patients without insight possess a more complex CBF disturbance. Simultaneously, our findings also incline to support the idea that the CBF aberrant in some specific brain regions may be the common neural basis of insight and AVH. Our findings support the mostly current hypotheses regarding AVH to some extent. Although our findings come from a small sample, it provide the evidence that indicate us to conduct a larger study to thoroughly explore the mechanisms of schizophrenia, especially the core symptoms of AVHs and insight.

## INTRODUCTION

Many previous studies, including our own, have confirmed that in schizophrenia patients with widespread and complex cerebral blood flow (CBF) disturbance, anti-psychotics can normalize the aberrant CBF [1–15]. These studies suggest that in these patients, with decreased CBF always located in the prefrontal and anterior cingulate cortices along with increased CBF in the striatum, the CBF alterations in the thalamus and temporal cortex showed that hyper or hypo CBF can co-exist in these regions, CBF alterations showed a complex aberrant pattern, and some CBF alterations are associated with the core clinical symptoms of schizophrenia [1–17]. These important findings led us to further explore the pathological mechanisms of schizophrenia, especially the mechanisms of the most common clinical symptoms, in order to find precise treatment targets and improve treatment strategy [17–20].

Auditory verbal hallucinations (AVH) are a core symptom of schizophrenia [16]. According to a report by Hugdahl *et al*., nearly 60–80% of schizophrenia patients experience AVH, presenting as distinct voices consisting of conversing, commenting or imperative contents; these different types of AVH always made the patients feel distress and induced significant psychosocial impairment, even suicide [17–20]. In the last several decades, many studies adopted diverse methods to investigate the mechanisms and target treatment of AVH and established some hypotheses to explain the mechanisms of AVH. For example, the unstable memories hypothesis suggests that AVH might be caused by the intrusion and unintended activation of memories [21]. The source monitoring hypothesis suggests that AVH are caused by deficits in the processing system of self-monitoring and reality discrimination; subsequently, some internal events, such as thoughts, inner speech and actions which lost self attributes, induce the vast difficulties in self-recognition [22]. The inter-hemispheric miscommunication hypothesis suggests that the synchrony increase between bilateral auditory regions contributes to the occurrence of AVH [23]. The top-down effect and bottom-up predictions hypothesis suggests that AVH may be attributed to the disturbance interaction between the bottom-up sensory processing system and the top-down processing system [24]. Four years before, Ford and Hoffman proposed the hypothesis of hybrid models of AVH, which suggested that the neural mechanism of AVH is through corticostriatal network hyperactivity and whereby otherwise nascent activity can be mixed into the consciousness [25]. Hugdahl *et al*. summarized the previous studies and proposed that AVH are likely initiated from temporal lobe neuronal hyper-activation which draws focus inward and are not inhibited by frontal lobe hypo-activation. In addition, this hypothesis also proposed that generated AVH are sustained through aberrant glutamate and likely mediated by the gamma-amino-butyric-acid transmitter. These transmitters may be the pharmacological target for AVH treatment [26].

Most of the abovementioned hypotheses explain the mechanisms of AVH from different perspectives and provide important and useful information for us understand the mechanisms of AVH. However, insight is also a key characteristic of schizophrenia; lacking or having insight can have a distinctly different influence on the clinical remission of schizophrenia [27, 28]. According to previous studies, insight is mediated by many brain networks, such as a self-monitoring network, and it is also involved in the occurrence of AVH [29]. Self-reflection networks are also related to insight [30], and the inferior frontal gyrus, anterior insula, and inferior parietal lobule, and ventromedial prefrontal cortex are all involved in the mediation of insight in schizophrenia [30]. Recently, Xavier *et al*. reported that the prefrontal and cingulate cortex, the precuneus, inferior parietal lobule and hippocampus form the neural basis of insight [31]. Unfortunately, to the best of our knowledge, rare studies reported the insight associated CBF alterations and few studies have been conducted to investigate the CBF alterations difference beween the AVH-experiencing schizophrenia patients with and without insight.

A review of the literature indicates that AVH and insight were mediated by some common brain regions and these regions’ neural activity reciprocation with each other [22–31]. However, to the best of our knowledge, no one has yet studied the brain characteristics of the AVH-schizophrenic patients with and without insight. In our previous study, we found that specific alterations of AVHs in schizophrenia patients are related to both the CBF increases in the auditory and striatal areas and the rCBF reductions in the visual and parietal areas [32]. Based on previous studies and our previous findings, we conduct an exploratory study to investigate the CBF aberrant differences in AVH-schizophrenia patients with and without insight using the 3D pseudo-continuous ASL (pcASL) technique,. We hypothesized that the pattern of CBF alterations of AVH-experiencing schizophrenia patients with insight will be different than the alterations of patients without insight, simultaneously, we aslo hypothesized there is a reciprocal action relationship existed between AVH-related brain network and insihgt-related brain network. These findings will enrich our understanding of the symptom-specific neural mechanisms of schizophrenia.

## RESULTS

### Demographic characteristics

Demographic data for the participants are shown in Table 1. The two patient groups had no significant differences in age, antipsychotic dosages, duration of illness and PANSS score. The severity of AVH was significantly different between the two patients groups. In addition, the AVH-schizophrenic patients without insight had higher auditory hallucination rating scale (AHRS) scores, indicating that the AVHs are more severe in these patients.

**Table 1:** Demographic characteristics of the participants

Characteristics	AVH-schizophrenic patients without insight patients *N* = 9	AVH-schizophrenic patients with insight patients *N* = 9	Healthy controls *N* = 9	t/F/Χ^2^	*p*
Age, years: mean (s.d.)	33.6 ± 11.1	33.4 ± 10.2	33.6 ± 11.2	0.001	0.999
Gender, femalen/male: n	3/6	7/2	5/4	3.600	0.058
Illness duration, years: mean (s.d.)	11.4 ± 6.4	11.2 ± 9.8		0.057	0.955
Antipsychotic dosage, mg/d: mean (s.d.)	374.07 (173.82)	375.18 (201.16)		0.011	0.992
PANSS, mean (s.d.)	70.3 (14.9)	70.4 (23.0)		0.012	0.990
AHRS, mean (s.d.)	6.77 (2.86)	4.44 (0.52)		2.405	0.029

Note: Antipsychotic dosages are reported as chlorpromazine equivalents calculated based on clinically equivalent dosing estimates.

### CBF differences across groups

**Figure 1:**
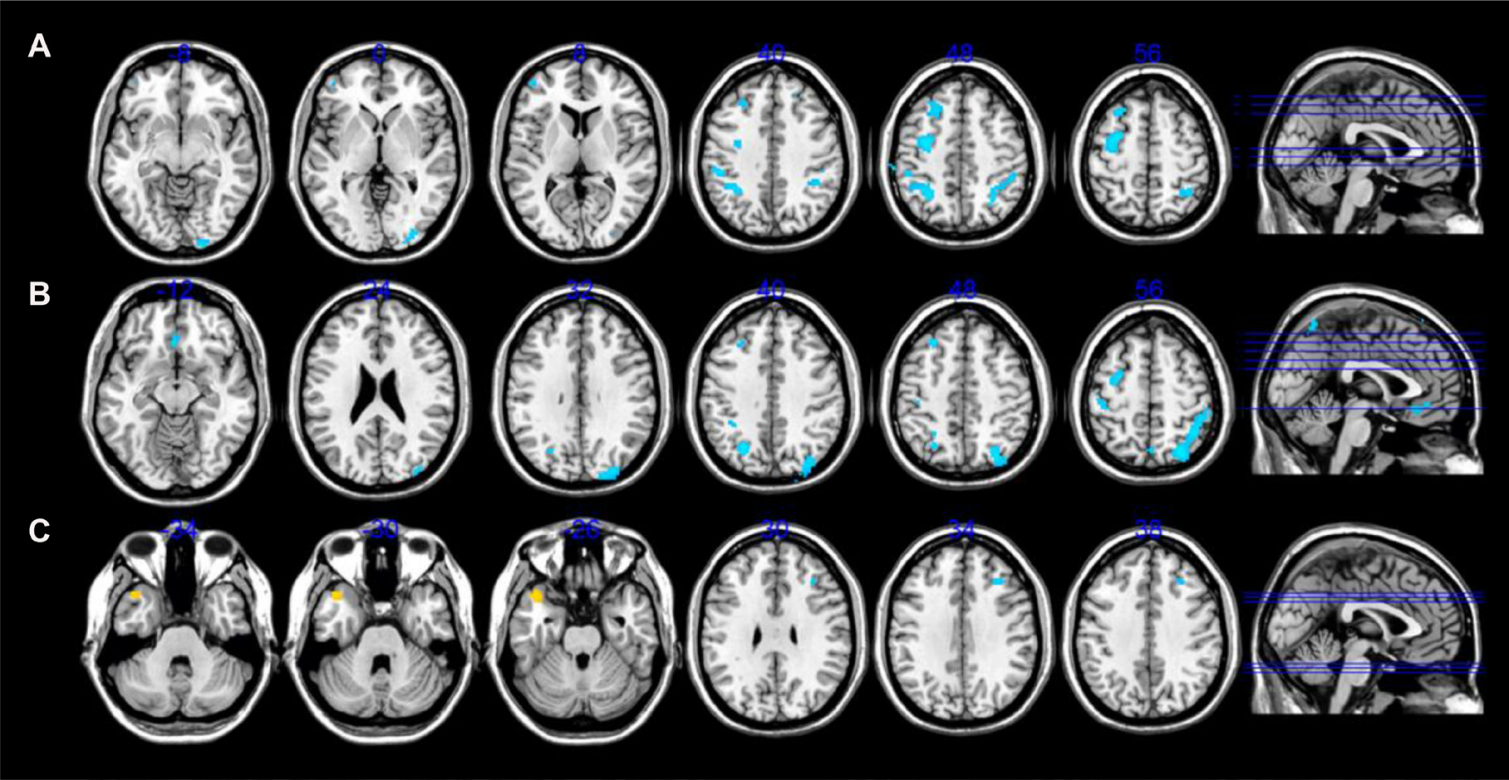
CBF alterations differences among AVH-schizophrenic patients without insight, with insight and healthy controls

Compared to healthy controls, AVH-schizophrenic patients with insight exhibited significantly decreased CBF, primarily located in the middle occipital gyrus, middle frontal gyrus, inferior parietal lobule, and left precentral regions (Figure 1A). However, no increase in CBF was found in AVH-schizophrenic patients without insight. In addition, in AVH-schizophrenic patients without insight, decreased CBF was found mainly in the superior parietal lobule, postcentral gyrus, inferior parietal lobule, middle frontal gyrus, and left precentral regions (Figure 1B). No increase in CBF was found in this group. Compared to AVH-schizophrenic patients with insight, AVH-schizophrenic patients without insight exhibited increased CBF located in the left temporal pole and decreased CBF located in the right middle frontal gyrus (Figure 1C). All the above differences are adjusted with an FDR correction, *P* < 0.05. The value of the CBF in the aberrant brain regions please see Table 2.

**Table 2:** The value of the CBF in the aberrant brain regions

Patients Group	Brain regions	CBF Pseudo-*T* value
AVH schizophrenia patients with complete insight; compared to healthy controls	Occipital Gyrus	−4.28
	Middle Frontal Gyrus	−4.13, −4.31, −4.43(Three cluster)
	Inferior Parietal Lobule	−5.02, −4.22, −4.68 (Three cluster)
	Left Precentral Gyrus	−5.45
AVH schizophrenia patients without complete insight; compared to healthy controls	Superior Parietal Lobule	−5.51
	Postcentral Gyrus	−4.74
	Inferior Parietal Lobule	−5.36
	Middle Frontal Gyrus	−4.9
	Left Precentral Gyrus	−4.9
AVH schizophrenia patients without complete insight;	Left temporal Pole	4.08
compared to the patients with complete insight	Right Middle Frontal Gyrus	−3.01

### Relationship between CBF and AVH severity in AVH groups

As in our previous study [32], we did not find any correlation between the CBF alterations and ARHS scores in the schizophrenia patients with or without insight.

## DISCUSSION

In the present study, we found that AVH-schizophrenia patients with and without insight demonstrated decreased CBF in the pivotal brain regions (occipital gyrus, frontal gyrus, inferior parietal lobule, precentral regions, superior parietal lobule, postcentral gyrus, middle frontal gyrus). These findings mostly align with our previous findings [32], although there are some inconsistencies. We found only decreased CBF located in the aforementioned brain regions, which compose the pivotal components of the language and auditory network, self-monitoring related network, visual network, self-reflection network and sensorimotor network and participate in top-down and bottom-up regulation [33]. The decreased CBF in these brain regions indicates that information processing ability is impaired, subsequently causing brain function disturbance, which manifests as a wide variety of clinical symptoms (including insight) especially in the patients of schizophrenia [1, 34–40]. Our findings incline to support that all these networks participate in the modulation of insight and AVH, the AVH related brain networks interacted with insight related brain network. Taken together, the CBF alterations in these regions represent the possibility that there is maybe a neural basis of the AVH and insight of schizophrenia. Additionally, these findings also incline to support the idea that insight and AVH have an influence on each other.

The most interesting finding in our present study is that AVH-schizophrenia patients without insight were more likely to have increased CBF in the left temporal pole and decreased CBF in right middle frontal gyrus when compared to AVH-schizophrenia patients with insight. These novel findings suggest that AVH-schizophrenia patients without insight possess a more complex CBF disturbance. Although this finding comes from a very limited sample, it provides an important clue for further investigations into the mechanisms of sub-group schizophrenia patients according to specific core symptoms. A large number of studies have already confirmed that temporal regions play a key role in the occurrence of AVH, and most of these studies identified that hyper-activity in the temporal regions is related to the occurrence of AVH. This point is accepted by many of the AVH-related hypotheses [26, 19, 33]. Similarly, the right middle frontal gyrus is a component of the central executive network (CEN), which participates in the mediation of many circuits and networks, such as the default network, the salience network and emotional processing circuit, the memory processing circuit, the language processing circuit, the attention processing and self-monitor processing circuit, etc. [40–42]. This region also plays a pivotal role in the occurrence of AVH; the hypo-activity of the CEN will loosen the control of hyper-activity of the temporal regions, hence, potentially causing the AVH [23, 25, 26, 33, 43, 44]. The decreased CBF in the right middle frontal gyrus and increased CBF in the left temporal pole indicated complex reciprocal action between different brain networks and inter-hemisphere communication disturbance [23, 25, 26, 37, 47, 48]. This finding indicates the need for further investigation into the sub-group of AVH schizophrenia patients in order to help further understanding of the pathological characters of the specific core symptoms of schizophrenia.

## LIMITATION

Although the severity of AVH is higher in patients without insight than patients with insight, we did not find that CBF correlated with the AHRS scores, as in our previous study. Currently, we cannot explain this inconsistent finding with certainty. However, we think some factors can explain this phenomenon. First, if CBF alteration is not associated with AVH,it is maybe due to a quality index, but this hypothesis needs further study. Second, all the patients have chronic schizophrenia and have many potentially confounding factors, which may require further study by enrolling drug-naive first episode patients in order to eliminate these confounders. Third, although we used age matching and assessed both illness duration and illness severity, we did not control for gender, which may be an influencing factor [45]. Fourth, although the illness severity is well matched, we know that some core symptoms of schizophrenia interact with one another. For example, delusion always influences hallucination, making the latter the most difficult factor to control. In sum, a well-designed study controlling for these confounding factors to the maximum extent possible is necessary. However, while this study would be time consuming and exhausting work, it could provide detailed descriptions of the pathological characteristics of schizophrenia and provide precise treatment targets. Researchers from around the world would likely be required in order to it complete this formidable task.

## CONCLUSIONS

The most interesting finding in our present study shows that AVH-schizophrenia patients without insight presented with increased CBF in the left temporal pole and decreased CBF in right middle frontal gyrus when compared to AVH-schizophrenia patients with insight. These novel findings suggest that AVH-schizophrenia patients without insight possess a more complex CBF disturbance. Simultaneously, we found that AVH-schizophrenia patients with and without insight all demonstrated decreased CBF in the pivotal brain regions, including the occipital gyrus, frontal gyrus, inferior parietal lobule, precentral regions, superior parietal lobule, postcentral gyrus, and middle frontal gyrus. Taken together, the CBF alterations in these regions represent the possibility that there is maybe a neural basis of AVH and insight synptoms in schizophrenia. Morover, our above findings incline to support the idea that the CBF aberrant in some specific brain regions may be the common neural basis of insight and AVH. Our findings support the mostly current hypotheses regarding AVH to some extent, although none one special hypothesis is completely supported by our findings. Notably, although our findings come from a small sample, they provide the evidence that indicate us to conduct a well-designed, larger study to thoroughly explore the pathological mechanisms of schizophrenia and its core symptoms.

## MATERIALS AND METHODS

### Participants

Based on our previous study which explored the AVH-specific CBF alterations [32], we selected 9 schizophrenia patients with complete insight and 9 schizophrenia patients without complete insight (because the sub-group sample is very difficult to enroll. To ensure comparability, we excluded patients with incomplete insights. The two groups of patients are well matched in age, dosage of anti-psychotic agents, illness duration and illness severity (as measured by PANSS scores). The age of healthy controls (*n* = 9) were well matched. However, due to sample limitations, the gender was not well matched. In analysis, age and gender were used as covariants. The inclusion and exclusion criteria for all participants were similar to our previous study [32]. The insight in this study is referring to the schizophrenia patients who have complete insight of his/her disease, including having the insight of his/her schizophrenic symptoms, such as delusions, hallucinations, thought insertions, etc. The insight was diagnosed by two professional psychiatrists according to an insight assessment scale [46].The Medical Research Ethics Committee of Tianjin Mental Health Center approved this study. After receiving a complete description of this study, written informed consent was obtained from every participant.

### MRI data acquisition

The MRI scans were performed by a 3.0-Tesla MR system (Discovery MR750, General Electric, Milwaukee, Wisconsin, USA). Comfortable and tight foam padding was added to control the head motion. Earplugs were provided to minimize scanner noise. The resting-state perfusion imaging was completed by a pseudocontinuous ASL sequence with a 3D fast spin-echo acquisition and background suppression. The parameters were as follows: TR = 4886 ms, TE = 10.5 ms, post-label delay 2025 ms, spiral in readout of eight arms with 512 sample points; flip angle, 111°; FOV = 240×240 mm; reconstruction matrix, 128×128; 40 axial slices; no gap; slice thickness, 4 mm. Number of excitations was 3 and in-plane resolution was 1.9×1.9 mm. The total acquisition time for the resting-state pcASL scan was 284 s. During the scans, all participants were asked to keep their eyes closed, relax and move as little as possible, and think of nothing in particular but not fall asleep. All images were visually inspected to ensure that only images without visible artefacts were included in subsequent analyses.

### CBF calculation

The CBF calculation method was similar to our previous study [32]. The pcASL difference images were calculated by a single compartment model [47] after the subtraction of the label images from the control images. The maps of CBF were derived subsequently from the ASL difference images and the proton-density weighted reference images [48]. Statistical parametric mapping (SPM8) [49] was used to co-register the CBF images of the 9 healthy controls to a PET-perfusion template in the MNI space using non-linear transformation. The standard CBF template of the MNI was referred to as the mean co-registered CBF image for the 9 healthy controls. The CBF images of all subjects were co-registered to the standard CBF template of the MNI and re-sampled to a voxel size of 2×2×2 mm. Non-brain tissue was removed from each co-registered CBF map and spatially smoothed with a Gaussian kernel of 8×8×8 mm full-width at half maximum (FWHM). We normalized the CBF of each voxel by dividing the mean CBF of the whole brain [50].

### Statistical analysis

Considering the limitation of the small sample, we performed group differences in CBF between any two groups (AVH-schizophrenic patients without insight vs. healthy controls, AVH-schizophrenic patients with insight vs. healthy controls, AVH-schizophrenic patients without insight vs AVH-schizophrenic patients with insight) using statistical non-parametric mapping (SnPM13, http://warwick.ac.uk/snpm.), which provides an extensible framework for non-parametric permutation tests based on the general linear model, and pseudo *t*-statistics for independent observations and can reduce the influences to the maximum which caused by the little sample [51] Pseudo voxel-level two-sample *t*-tests based on SnPM were applied to between-group analyses with age, gender, illness duration and antipsychotic dosages as covariates (permutation test *n* = 10000, *p* < 0.001, cluster size >100 for the following groups: AVH-schizophrenic patients without insight vs. healthy controls, AVH-schizophrenic patients with insight vs. healthy controls; cluster size >50 for AVH-schizophrenic patients without insight vs AVH-schizophrenic patients with insight). In order to explore the relationship between CBF alterations and AVH severity, a multiple regression analysis was used in the two AVH groups with regions exhibiting significantly different CBF aberrance compared with the healthy control groups. In this analysis the gender, age, illness duration and antipsychotic dosages were considered nuisance covariates.

SPSS 19.0 statistical analysis software (SPSS, Inc., Chicago, IL, USA) was adopted for all statistical analyses of all data from the three groups. The statistical analyses were performed using two independent sample *t*-tests, square test and variance analysis. *P* < 0.05 was considered to indicate a statistically significant difference. The data are presented as the mean ± standard deviation.
